# Soot elimination and heat recovery of industrial flue gas by heterogeneous condensation

**DOI:** 10.1038/s41598-020-59833-3

**Published:** 2020-02-19

**Authors:** Liang Ma, Zhihuang Zhao, Chengcheng Tian, Hualin Wang, Yi Liu

**Affiliations:** National Engineering Laboratory for Industrial Wastewater Treatment, East China University of Science and Technology, Shanghai, 200237 P.R. China

**Keywords:** Atmospheric chemistry, Pollution remediation

## Abstract

Industrial flue gas systems include fine soot and high-temperature vapor. The continuous emission of the flue gas not only causes fine particulate pollution but also wastes considerable heat energy. Separating soot and purifying flue gas are of great significance for industrial conditions and environmental protection. In this paper, the process of cyclone soot elimination and waste heat recovery by heterogeneous condensation were coupled for the first time. The effects of the flue gas material system and separation operation parameters on the cyclone soot elimination efficiency and heat transfer efficiency were systematically investigated through unit experiments and industrial side-lines. Additionally, the mechanism of enhanced cyclone soot elimination by heterogeneous condensation was also theoretically explored. The experimental results show that the corresponding maximum cyclone heat transfer efficiency and soot elimination efficiency of the Ф40 mm cyclone separator are 42.1% and 89.2%, respectively, while the Ф80 mm cyclone separator can attain an elimination efficiency of 91% and a maximum increase of 67.3% for the heat transfer efficiency, as indicated by the industrial side-line. During the process of cyclone soot elimination and heat recovery by heterogeneous condensation, the heterogeneous condensation caused by heat transfer increases the quality difference between the flue gas molecules and soot droplets, thus improving the cyclone separation efficiency of soot.

## Introduction

With the continuous optimization of China’s energy structure, the energy consumption and energy utilization rate in China have been increasing. The energy utilization rate of developed countries is generally over 43%, while that of China is only approximately 33%. At least 50% of the energy in industry is directly discharged in various forms of waste heat. Among them, a large quantity of industrial incineration flue gas containing fine soot and waste heat is directly exhausted, resulting in a substantial waste of resources and environmental pollution^1^. Waste heat recovery from flue gas can greatly improve the energy utilization efficiency and reduce production costs. The boiler efficiency increases by 1% for every 10–15 °C reduction in the flue gas temperature^2–4^. The removal of the flue gas and soot, especially fine soot (<2.5 microns), can significantly alleviate growing haze and improve the flue gas quality^5^. At present, high-temperature flue gas has a high thermal energy grade, less difficult utilization and better waste heat recovery. Medium- and low-temperature flue gas exists in power plants and petrochemical enterprises; these gases are discharged with a large amount of soot and have a low waste heat recovery efficiency. Therefore, it is particularly important to develop waste heat recovery equipment with a simple structure and a low initial investment cost. The reasonable and efficient recovery of waste heat resources in low- and medium-temperature flue gas is of great significance for energy savings, consumption reduction and energy efficiency improvement^6–8^.

Cyclone separators are common equipment for soot elimination in chemical production processes^9,10^. Soot-containing flue gas enters the cylinder part of the cyclone separator tangentially and begins to whirl. Soot is thrown to the cylinder wall by centrifugal forces, loses kinetic energy, and falls into the soot collector. When dealing with a large amount of the flue gas, several cyclone separators are connected in parallel to improve the capacity and efficiency of the cyclone separators. The cyclone separator is characterized by its simple fabrication, especially suitable for flue gas with a larger particle size and higher soot concentration. Some scholars have studied the high-temperature soot elimination of cyclone separators, which lays a foundation for soot elimination by cyclone separators with heat recovery at high temperatures.

It was found that the higher the temperature of the flue gas is and the smaller the particle size of soot is, the better the heat transfer performance of the cyclone separator is, but the thermal conductivity of soot in the flue gas had little effect on the heat transfer performance of the cyclone separator^11^. Furthermore, the soot elimination efficiency of cyclone separators decreases with increasing flue gas temperature and pressure drop^12^. The effects of the particle size, inlet velocity and particle arrangements at the entrance on improving the separation efficiency of cyclones have also been studied^13,14^. The experimental study theoretically analyzed the soot elimination performance of cyclone separators and simulated the heat transfer performance of high-temperature flue gas in cyclone separators^15^. By studying the effect of water vapor on the electrostatic precipitator, it was found that water vapor can enhance the soot elimination efficiency, especially soot with particle sizes less than 2 µm^16^.

Heterogeneous condensation can enhance the efficiency of soot elimination and the recovery of latent heat in the flue gas and has the advantages of a high soot elimination efficiency and relatively small resource consumption. These studies have been conducted in-depth.

A wet electro-cyclone was designed and tested with high efficiency for long-term operation, and the total collection efficiency was maintained at 93.0% after 14 days of continuous operation^17^. The soot elimination efficiency of 0.8 μm ferric oxide and titanium dioxide by heterogeneous condensation was over 95%, and the pressure drop was low^18^. Both hydrophilic and hydrophobic particles grew rapidly in a supersaturated environment^19^. The particle’s prolapse performance was directly proportional to the particle size and wettability in a saturated environment^20^. The fluid flow characteristics of particles were examined in the boundary layer of a cyclone separator^21,22^.

Although the above research has laid a foundation for the application of cyclone separators and soot elimination by heterogeneous condensation, there is no relevant research on cyclone separators used in soot elimination and heat recovery by heterogeneous condensation. Based on the soot elimination and heat transfer performance of the cyclone separator, this paper first explores the effect of key factors such as the flue gas volume, soot concentration, flue gas temperature and cooling medium volume on the heat transfer efficiency, soot elimination efficiency, pressure drop, heat transfer coefficient and flue gas temperature drop. In addition, a self-designed cyclone separator is used as a heat exchanger tube for industrial side-lines. The cooling medium flows outside the cyclone tube, and the rotating flue gas in the cyclone separator exchanges heat through the tube wall. When the flue gas temperature decreases below the saturated vapor temperature of the water vapor, the saturated vapor condenses small droplets with soot particles as condensation nodules, releases latent heat and increases the efficiency of waste heat recovery. At the same time, under the action of centrifugal force, soot droplets will further capture soot particles when moving toward the sidewall of the cyclone separator, which can enhance the separation efficiency of soot. Therefore, research on soot elimination and heat recovery by heterogeneous condensation has innovative significance.

## Results

### Lab-scale experiment for heat transfer efficiency optimization

**Figure 1:**
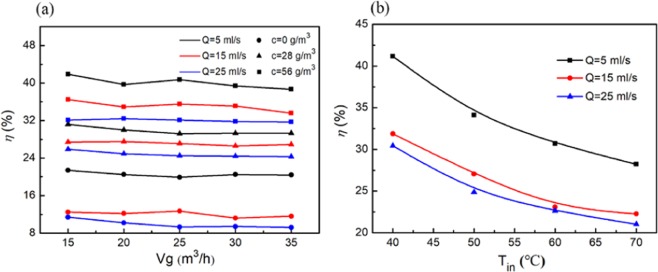
Effect of flue gas volume, flue gas temperature, soot concentration and cooling medium volume on heat transfer efficiency.

The gas volume, soot concentration and cooling medium volume are important factors that affect the heat transfer efficiency. The temperature of the cooling medium in the experiment was based on the ambient temperature (20 °C). To explore the influence of the flue gas volume on the heat transfer efficiency, the flue gas temperature was adjusted to 40 °C, the soot concentrations were 0, 28, and 56 g/m3, and the cooling medium volumes were 5, 15 and 25 ml/s. It can be seen from Fig. 1(a) that in the same experimental device, the heat transfer efficiency of pure vapor is lower than that of the soot-containing vapor. When the soot concentration is 0 g/m^3^, the maximum heat transfer efficiency can reach 21.4%, while the maximum heat transfer efficiency can reach 41.9% when the soot concentration is 56 g/m^3^. This result indicated that the soot-containing flue gas system has a better effect on waste heat recovery than that of the pure vapor system. As known, soot particles would surely be considered the adhering carrier for vapor-water molecules in the process of vapor condensation. Nucleation efficiency curves showed that adhering carrier can accelerate vapor condensation^23^, and therefore condensation acceleration can produce more droplets per unit of time due to high-speed rotation and frequent collision in the cyclone separator. Clusters accelerate the growth of small droplets, thus increasing the condensation amount of vapor and the heat released by condensation per unit time.

Under the same cooling medium volume, the heat transfer efficiency of the cyclone separator decreases slowly with increasing cooling medium volume. When the cooling medium volume is 5 ml/s, the maximum heat transfer efficiency is approximately 40%, and when the cooling medium volume is 25 ml/s, the maximum heat transfer efficiency is approximately 30%. The main reason is that when the liquid volume is small, the cooling medium stays in the jacket for a long time, which improves the recovery of waste heat. The flue gas temperature is an important factor for heat transfer efficiency. As shown in Fig. 1(b), the heat transfer efficiency of the cyclone separator decreases with increasing temperature when the flue gas volume is 35 m^3^/h, the soot concentration is 56 g/m^3^, and the cooling medium volumes are 5, 15 and 25 ml/s. When the flue gas temperature increases from 40 °C to 70 °C, the heat transfer efficiency of the cyclone separator decreases with increasing flue gas temperature, and the maximum is 42.1% of the temperature at 40 °C. The reason is that when the cooling medium volume is constant, the heat absorption temperature of the cooling medium increases rapidly, resulting in a reduction in the heat transfer coefficient and the heat transfer efficiency.

**Figure 2:**
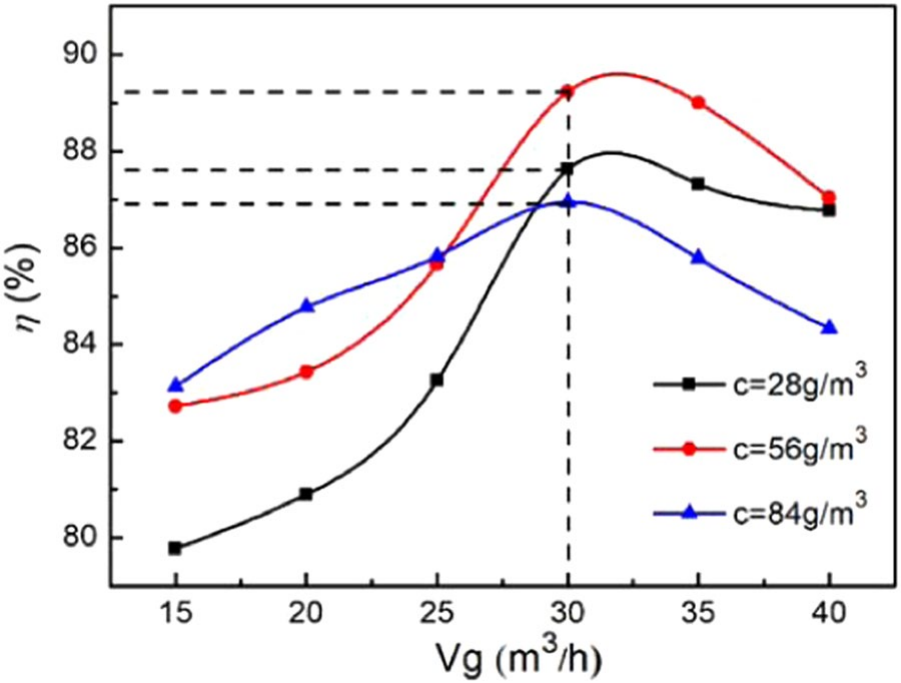
Effect of flue gas volume on soot elimination efficiency.

As shown in Fig. 2, under the condition that the flue gas is 40 °C and the cooling medium is 20 °C with a volume of 5 ml/s, the variation in the soot elimination efficiency with a flue gas volume of 15–40 m^3^/h was studied by adjusting the soot concentrations of 28, 56 and 84 g/m^3^. Under the same soot concentration, the soot elimination efficiency of the cyclone separator increases first and then decreases rapidly with increasing flue gas flow. When the flue gas volume is 32 m^3^/h, the soot elimination efficiency reaches the maximum. It can be explained that when the volume is small, the rotating centrifugal force in the cyclone separator increases with increasing volume, and the soot can be moved to the sidewall of the cyclone separator and separated. When the soot elimination efficiency reaches a maximum, the resistance in the cyclone separator increases sharply with a further increase in the volume, and even the phenomenon of rebound and back mixing of the soot particles arises, which reduces the soot elimination efficiency. The maximum soot elimination efficiencies of the different soot concentrations are 87.6%, 89.2% and 86.9% at concentrations of 28, 56 and 84 g/m^3^, respectively.

### Results of the industrial side-line test

**Figure 3:**
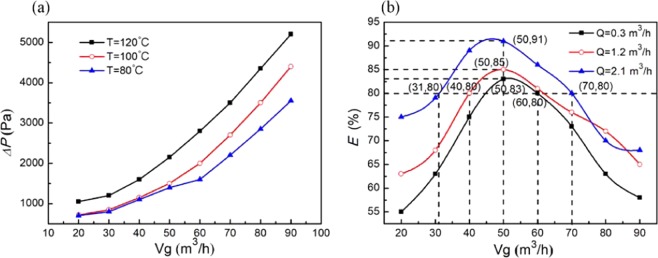
Effect of flue gas volume on pressure drop and soot elimination efficiency.

When the cooling medium volume is 2.1 m^3^/h and the flue gas temperature is 120, 100 and 80 °C, the pressure drop between the inlet and outlet of the experimental system is measured. As shown in Fig. 3(a), the pressure decreases with increasing volume, and the rate of increase accelerates. Under the same gas volume, the pressure decreases with increasing gas temperature. Usually, the pressure loss of the cyclone separator is mainly caused by pipe overflow and swirling, but the higher the temperature of the flue gas is, the greater the viscosity of the soot in the flue gas is and the greater the frictional resistance between the flue gas and the cyclone separator wall is, resulting in decreased pressure.

As shown in Fig. 3(b), when the gas temperature is 120 °C, the soot elimination efficiency increases first and then decreases rapidly with increasing gas volume, and the soot elimination efficiency reaches a maximum when the gas volume is approximately 50 m^3^/h. At the same volume, the separation efficiency increases with an increase in the cooling medium volume, which is mainly because of the faster condensing high-temperature flue gas. On the one hand, the precipitation of the condensate strengthens the soot separation efficiency, especially for fine soot (<0.3 micron). On the other hand, the precipitation of the condensate increases the tangential resistance of the gas along the wall of the cyclone separator tube, and the tangential velocity of the gas and the separation efficiency decrease. When the cooling medium volume is 2.1 m^3^/h and the flue gas volume is 50 m^3^/h, the maximum efficiency of soot elimination is 91%. Under the same volume and different cooling medium volumes, the maximum range of the soot elimination efficiency is less than 20%.

**Figure 4:**
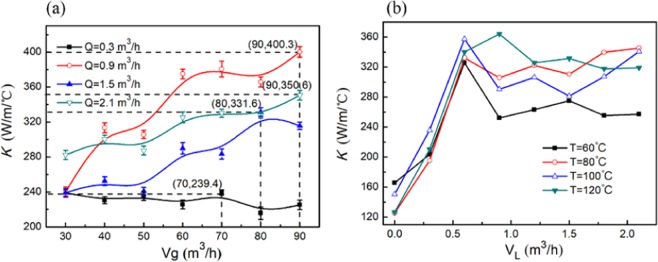
Effect of cooling medium volume, flue gas temperature, flue gas volume on heat transfer efficiency.

The heat transfer coefficient, K, is an important parameter that reflects the efficiency of the waste heat recovery. For the finalized system, the physical properties and volume of the fluids on both sides of the wall are important factors for the heat transfer coefficient, and the decrease in the flue gas temperature can intuitively reflect the effect of the waste heat recovery. The relationship between the heat transfer coefficient, K, and the volume of the flue gas and cooling medium was given. As shown in Fig. 4(a), when the flue gas temperature is 120 °C, the heat transfer coefficient increases with increasing flue gas volume, and when the volume of the cooling medium is 0.9 m^3^/h, the maximum increase is 67.3%. As shown in Fig. 4(b), when the flue gas volume is 80 m^3^/h and the coolant volume increases from 0.3 m^3^/h to 2.1 m^3^/h, the heat transfer coefficient increases rapidly with increasing coolant. When the coolant volume increases to 0.6 m^3^/h, the heat transfer coefficient remains unchanged with increasing coolant volume, and the higher the flue gas temperature is, the greater the heat transfer coefficient is. The difference is mainly because the thermal resistance on the flue gas side is smaller than that on the cooling medium side, and the flue gas rotates centrifugally in the swirl tube. With increasing flue gas volume, the renewal frequency of the boundary layer on the flue gas side of the cyclone separator increases, the thermal resistance decreases, and the heat transfer rate increases^24^. In addition, because the cyclone separator is oriented vertically, the vertical laminar flow increases the disturbance of the flue gas near the wall. The downward and outward swirling flow in the cyclone separator reduces the thickness of the condensate on the wall of the tube and continuously scours the wall of the cyclone separator to prevent fouling, enhance heat transfer and improve the heat transfer coefficient on the flue gas side^25^. The heat transfer coefficient of the heat exchanger increases first and then remains constant with an increase in the volume of the cooling medium. Under the experimental conditions, when the volume of the cooling medium is less than 0.6 m^3^/h, the Reynolds number on the cooling medium side increases with an increase in the volume of the cooling medium^26,27^.

### Principle of soot elimination by heterogeneous condensation

When the flue gas temperature decreases rapidly, an oversaturated vapor environment is formed. Water vapor is condensed on the fine soot particles, which act as condensation nuclei, and the mass and size of the particles increase. In the separation flow field, soot is trapped by condensation droplets. Vapor condenses on the surface of the particles via two processes: heterogeneous nucleation and condensation. In the heterogeneous nucleation process, the supersaturation of water vapor should be higher than the critical supersaturation, which mainly depends on the particle size and shape, surface physical and chemical properties and water vapor properties^28^.

**Figure 5:**
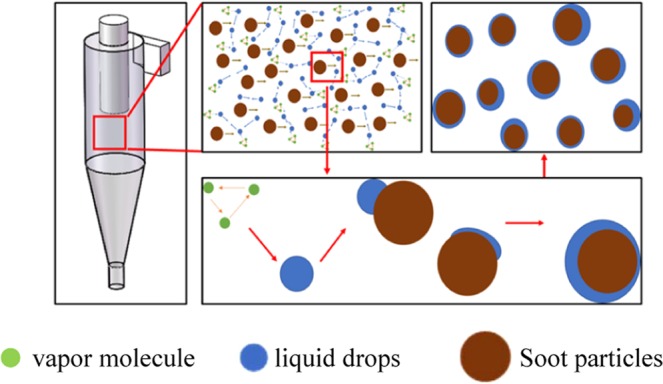
Schematic diagram of heterogeneous condensation.

When the vapor contacts the condensation surface, it condenses at the core of the condensation nodule, and tiny droplets are formed. The core of the condensation nodule is covered by small droplets. At the same time, the vapor condenses on the surface of the small droplets, and the small droplets continuously grow. The droplet gradually grows up and begin to merge with each other, and then slips down when its gravity is greater than the adhesion force. The corresponding droplet size at this critical time point is defined as the slip size. When the droplet size reaches its slip size, the droplet begins to slip away from the condensation surface as shown in Fig. 5.

In the cyclone separator, water molecules in the vapor collide combine with each other to form clusters under the action of a high-speed rotating gas flow field^29^. The high-speed rotating gas flow field can not only provide the centrifugal force for gas-solid separation, but also accelerate the self-rotation of particles. It has been confirmed that the self-rotation of particles in a hydrocyclone can enhance the efficiency of removing pollutants in catalyst particles^30^. Based on this, a hydrothermal-hydrocyclone process has been proposed to enhance the management of the waste catalyst for residue oil hydro-treating^31^. When the vapor supersaturation exceeds the critical value, the vapor first accumulates on the surface of the particles, resulting in heterogeneous nucleation and condensation. The condensate droplet clusters are separated to the metal tube wall under centrifugal forces and eventually flow down into the soot collector to complete the collection of soot particles to achieve gas-solid separation. During this process, a large amount of latent heat emitted by vapor condensing is recycled.

## Discussion

**Figure 6:**
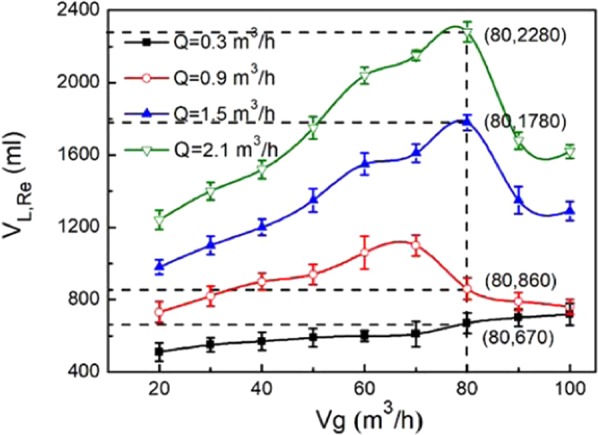
Effect of flue gas volume on separation efficiency.

With decreasing flue gas temperature, the water vapor in the flue gas gradually reaches supersaturation. When the flue gas temperature further decreases, the saturated water vapor condenses out with soot as condensation nuclei, and the water vapor is recovered in the form of condensed water. As shown in Fig. 6, when the temperature is 120 °C, the volume of the cooling medium is 0.3–2.1 m^3^/h, and the flue gas volume is 20–100 m^3^/h. The variation law of condensate recovery with the flue gas volume and cooling medium volume is obtained. The results show that the recovery of the condensate increases first and then decreases with increasing volume under different cooling medium volumes, and the faster the volume of the cooling medium is, the faster the increase or decrease in the condensate rate is. This behavior is because when the volume of the condensed water is low, the cooling effect is poor, and the amount of condensed water is low. With increasing volume of the cooling medium, the cooling effect is enhanced, and the water vapor in the flue gas easily condenses out. When the flue gas volume is less than 80 m^3^/h, the larger the flue gas volume is, the larger the water volume condensed from vapor per unit time is, and more condensed water is recovered. When the flue gas volume is greater than 80 m^3^/h, the residence time in the cyclone separator is too short to condense vapor. Under the same cooling medium volume, the recovery of condensed water increases first and then decreases with increasing gas volume, and the recovery of condensed water is the greatest when the gas volume is 60–80 m^3^/h. Under the same flue gas volume, the recovery of the condensate increases with increasing cooling medium volume.

The economic performance of cyclone separator is mainly measured by the change of energy in the process of heterogeneous condensation, which can be expressed by pressure drop and temperature drop. Energy balance and cost assessment are shown in Table 1, where *E*_*ΔP*_ is the energy consumed due to the pressure drop of separation, *V* is flue gas volume, *Δt*_*m*_ is logarithmic mean temperature difference, *E*_*ΔT*_ is the heat recovery from the heat transfer, *K* is the heat transfer coefficient, *A*_0_ is the total heat exchange area. It can be seen from Table 1 that energy consumed for separation is 0.5 kWh, energy recovered from heat transfer is 28.7 kWh and net energy recovery of the total gas-solid separation process accompanied by heterogeneous condensation is 28.2 kWh, which transmute to net profit is 2.933USD/h. It proves that the process can greatly reducing the power consumption, which is of great significance to reduce the cost and energy consumption.

**Table 1:** Energy balance and cost assessment.

No.	Items	Separation	Separation& Heat transfer	Unit
Energy balance (per hour)				
1	Energy consumed (separation) =${E}_{\varDelta P}=\frac{{H}_{1}\cdot \Delta p\cdot V}{D}$	−0.5	−0.5	kWh
2	Energy recovery (heat transfer) = $$\Delta {t}_{m}=\frac{\Delta {T}_{1}-\Delta {T}_{2}}{\mathrm{ln}(\frac{\Delta {T}_{1}}{\Delta {T}_{2}})}$$ $${E}_{\Delta T}=K\times {A}_{0}\times \Delta {t}_{m}$$$${E}_{\Delta T}=K\times {A}_{0}\times \Delta {t}_{m}$$	0	28.7	kWh
3	Net energy recovery = *No*.1 + *No*.2	−0.5	28.2	kWh
Cost calculation (per hour)				
4	Net profit = *No*.3 × 0.104*	−0.052	2.9328	USD

*The power price is the same as the announcement (2018) of state grid Wuhan power supply company.

## Methods

### Experimental

**Figure 7:**
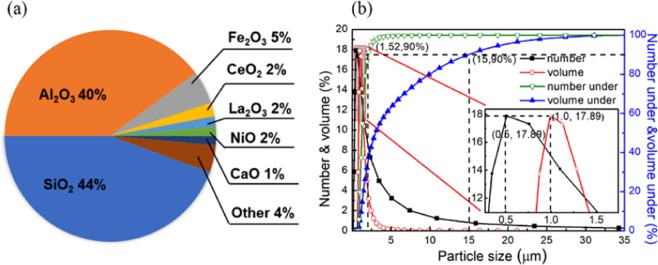
Soot composition and particle size distribution.

In the experiment, the incineration flue gas from the boiler of Wuhan Petrochemical Company was taken as the experimental object. The operating temperature of the flue gas was 60–160 °C, and the operating volume was 30–90 m^3^/h. The particle morphology and composition analysis showed (Fig. 7a) that O, Al, Si, and Fe are the main elements in the particles, while Ce, La, Ni, Ca, V, and S are the minor elements in the particles. The particulates are aluminosilicate minerals with moderate hydrophobicity, whose average particle size is 1.1 microns, and 90% of them are less than 1.8 microns (Fig. 7b). The bottom of the cylinder is connected with a conical soot collector for collecting separated soot and condensate water. The jacket wrapped outside the cyclone separator, the inlet and outlet of the cooling medium and the outlet and outlet of the flue gas are fixed on the stainless-steel frame. The power of the flue gas preheater is controlled by adjusting the regulator, and the different temperatures can be reached by preheating the simulated soot-containing flue gas. The influence of the temperature on the heat transfer efficiency and the soot elimination efficiency is studied.

**Figure 8:**
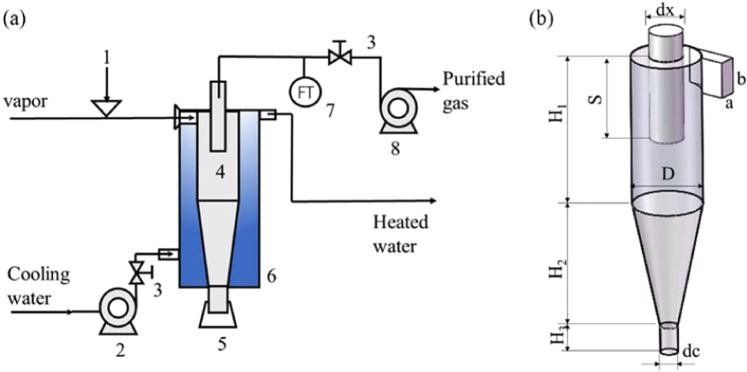
Experiment flow.

As shown in Fig. 8, after the jacket is filled with cooling medium, the fan is turned on. The flue gas is fully preheated in the flue gas preheater and then mixed with catalyst soot at the open manhole to form the high-temperature soot-containing flue gas. After the flue gas enters the cyclone separator, on the one hand, under the action of centrifugal forces, gas-solid separation is carried out in the cyclone separator, and the soot particles are separated. On the other hand, hot flue gas in the cyclone separator exchanges heat with the cooling medium outside through the metal tube wall. During the experiment, the flow direction of the flue gas is opposite to that of the cooling medium, which can improve the heat transfer efficiency. From the transverse section, the rotation direction of the cooling medium in the jacket is opposite to that of the flue gas in the cyclone separator. In the experimental device, the metal jacket at the cyclone separator is wrapped with insulation material, which reduces the heat exchange between the ambient temperature and the cyclone separator. Under the condition of no cooling medium in the jacket, the temperature of the flue gas at the inlet and outlet of the cyclone separator is almost unchanged.

### Industrial side-line

**Figure 9:**
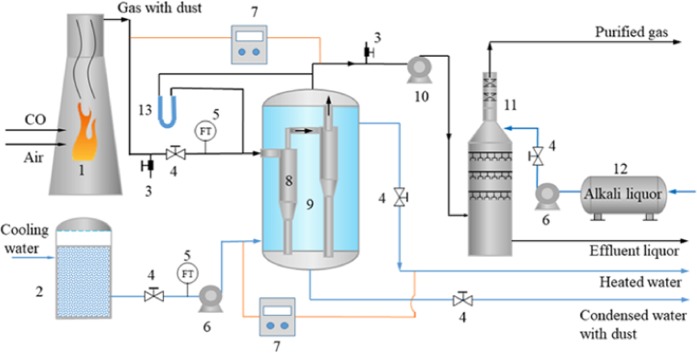
Flowchart of industrial side-line.

In this experimental system, the cyclone separator, tank and pipes are all made of 304 stainless steel, which has good heat transfer performance and corrosion resistance. As shown in Fig. 9, CO and flue gas are burned in a waste heat furnace, and the high-temperature flue gas containing soot generated by combustion is drawn into the cyclone separator (8) through a fan (10). At the same time, the cooling liquid in tank (2) enters tangentially from the bottom of the heat exchanger by using a centrifugal pump, and the soot-containing flue gas is centrifugally separated in the cyclone separator. The cooling medium outside the cyclone separator exchanges heat with the flue gas, resulting in a rapid decrease in the temperature of the flue gas, and the latent heat is released to form a saturated vapor environment. The water vapor changes phase by condensing on the fine soot particles acting as condensation nuclei. Meanwhile, under the action of centrifugal forces, soot droplets contact and collide with each other and then solidify, grow and separate. The rotational direction of the flue gas in the cyclone separator (8) is opposite to the entry direction of the cooling medium, which improves the heat transfer efficiency. The separated soot-containing condensate discharges from the bottom of the tank, and the flue gas enters the lower part of the desulfurization tower after heat exchange and soot elimination. Under the action of an anticorrosion pump (13), the alkali liquor (12) is conveyed to the top of the desulfurization tower (11) and sprayed downwards. The purified gas is discharged into the atmosphere. At the inlet and outlet of the flue gas and cooling liquid containing soot, thermometers are used to measure the temperatures of the flue gas and cooling medium. Flowmeters (5) measure the volumes of the flue gas and cooling medium, and a bypass valve (3) is used to regulate the volume and the concentration of the flue gas.

The cyclone separator used in this experiment reduces the diameter of the cylinder section, enhances the centrifugal effect and appropriately increases the length of the cone. A parallel guide plate at the inlet is added to stabilize the flue gas flow. The size parameters of the cyclone separator used in the experiment and industrial side-line are shown in Table 2. Meanwhile, the heat transfer efficiency can be expressed as:

1 $${\rm{\eta }}=\frac{\frac{1}{2}{q}_{mc}{c}_{c}({T}_{{\rm{c2}}}^{2}-{T}_{{\rm{c1}}}^{2})}{\frac{1}{2}{q}_{mc}{c}_{c}({T}_{{\rm{h1}}}^{2}-{T}_{{\rm{h2}}}^{2})}=\frac{{q}_{mc}{c}_{c}({T}_{{\rm{c2}}}-{T}_{{\rm{c1}}})({T}_{{\rm{c2}}}+{T}_{{\rm{c1}}})}{{q}_{mh}{c}_{h}({T}_{{\rm{h1}}}-{T}_{{\rm{h2}}})({T}_{{\rm{h1}}}+{T}_{{\rm{h2}}})}$$

**Table 2:** Parameters of cyclone separators used in the experiment and industrial side line (unit in mm unless cone angle *θ*).

	D	a	b	d_x_	d_c_	H_1_	H_2_	H_3_	S	I_n_	θ
Experiment	40	25	14	16	18	180	120	40	150	50	9°
Industrial side line	80	55	27	26	30	300	140	200	200	50	9°

In the formula, *q*_*mc*_ and *q*_*mh*_ represent the mass flow of cold and hot fluids, *C*_*C*_ and *C*_*h*_ are the constant pressure specific heat capacities of cold and hot fluids, *T*_*c1*_ and *T*_*c2*_ are the inlet and outlet temperatures of cold fluids, and *T*_*h1*_ and *T*_*h2*_ are the inlet and outlet temperatures of hot fluids, respectively. During the process of heat transfer, the reduction in the pyrolysis of hot fluids can be expressed as: $\frac{1}{2}{q}_{mh}{c}_{h}({T}_{{\rm{h1}}}^{2}-{T}_{{\rm{h2}}}^{2})$ and the increase in the pyrolysis of cold fluids can be expressed as: $\,\frac{1}{2}{q}_{mc}{c}_{c}({T}_{{\rm{c2}}}^{2}-{T}_{{\rm{c1}}}^{2})$^32^. In the system, the equation for the heat transfer efficiency is simplified to

2 $${\eta }_{h}=\frac{{T}_{c1}+{T}_{c2}}{{T}_{h1}+{T}_{h2}}$$

Furthermore, in the separation system of the cyclone separator, particulate soot consists of three parts: soot entering, soot captured and soot discharged. Then, the soot elimination efficiency of the cyclone separator can be expressed as

3 $${\rm{\eta }}=\frac{{M}_{c}}{{M}_{f}}=1-\frac{{M}_{e}}{{M}_{f}}$$

where *M*_*f*_ is the mass of inlet soot, *M*_*c*_ is the mass of the captured soot, and *M*_*c*_ is the mass of the escaped soot^33^.
